# Cardiovascular Manifestations Documented in Patients with Lyme Disease: Clinical Presentation, Management Strategies, and Outcomes

**DOI:** 10.3390/idr18030040

**Published:** 2026-04-27

**Authors:** Luis Antonio Cortes Islas, Priscila Mishelle Bartolo Gomez, Nora Denice Cuevas Obispo, Ayelen Xicohtencatl Muñoz, Lao Yuling Lopez Lucero, Juan Pablo Ramirez Hinojosa

**Affiliations:** 1 Division of Infectious Diseases and Epidemiology, Dr. Manuel Gea Gonzalez General Hospital, Calzada de Tlalpan 4800, Belisario Dominguez secc 16, Tlalpan, Mexico City 14080, Mexico; luisantoniocortes2000@gmail.com (L.A.C.I.); priscila.bartolog@alumno.buap.mx (P.M.B.G.); mc20cuon8484@facmed.unam.mx (N.D.C.O.); 2 Division of Basic Research, National Cancer Institute, San Fernando No. 22, Tlalpan, Mexico City 14080, Mexico; ayelenxm@gmail.com; 3 Emergency Division, Dr. Manuel Gea Gonzalez General Hospital, Calzada de Tlalpan 4800, Belisario Dominguez secc 16, Tlalpan, Mexico City 14080, Mexico; llopezl2404@alumno.ipn.mx

**Keywords:** Lyme disease, *Borrelia burgdorferi*, Lyme carditis, atrioventricular block, myocarditis, infectious endocarditis

## Abstract

Background/Objectives: Lyme disease is a tick-borne zoonosis caused by *Borrelia burgdorferi* that can affect multiple organ systems. Although cardiovascular involvement is considered uncommon, it may lead to severe and potentially life-threatening complications, particularly conduction disturbances and inflammatory cardiac conditions. This review aims to describe the spectrum of cardiovascular manifestations documented in patients with Lyme disease, focusing on clinical presentation, diagnostic approaches, management strategies, and reported outcomes. Methods: A narrative literature review was performed using PubMed, MEDLINE, and Google Scholar. Articles published between January 2000 and July 2025 in English or Spanish were screened. Eligible studies included original research articles, systematic and narrative reviews, case series, and case reports describing confirmed Lyme disease with cardiovascular involvement. A total of 30 studies were included. The available evidence was predominantly based on case reports and small case series, with considerable heterogeneity in study design, patient populations, and reported outcomes. Data on clinical manifestations, diagnostic methods, treatment strategies, and outcomes were extracted and synthesized. Results: Atrioventricular conduction disturbances were the most frequently reported cardiovascular manifestation, ranging from first-degree block to complete heart block, often presenting abruptly with syncope or bradycardia. Other reported manifestations included atrial and ventricular arrhythmias, myocarditis, pericarditis, myopericarditis, valvular endocarditis, aortitis, and vasculitis. Diagnosis relied on a combination of clinical suspicion, epidemiologic exposure, serologic testing, electrocardiographic monitoring, and cardiac imaging. Most patients were treated with antimicrobial therapy, commonly intravenous ceftriaxone followed by oral doxycycline, with temporary pacemaker support required in selected cases. Overall, clinical outcomes were favorable when treatment was initiated promptly. Conclusions: Cardiovascular involvement in Lyme disease, although infrequent, encompasses a broad clinical spectrum with potentially serious consequences. Early recognition, appropriate diagnostic evaluation, and timely antimicrobial therapy are essential to ensure reversibility of cardiac manifestations and favorable outcomes. However, the available evidence is limited by heterogeneity and the predominance of low-level-evidence studies.

## 1. Introduction

Lyme disease is a zoonosis transmitted by ticks of the genus Ixodes, primarily in temperate regions of the Northern Hemisphere, including North America, Europe, and Asia [1,2]. It is caused by *Borrelia burgdorferi*, a spirochete that, once inoculated into a human host, is capable of producing a wide variety of clinical manifestations [1].

Clinical presentation is usually divided into three stages: early localized, early disseminated, and late. In advanced stages, the infection can affect various systems, among them, the central nervous system, skin, joints, and, less frequently, the cardiovascular system [1,3,4]. Although it is estimated that only 1.5% to 10% of untreated patients develop Lyme carditis, its occurrence can have serious consequences [5,6].

Cardiovascular manifestations of Lyme disease are diverse, most commonly affecting the cardiac conduction system. Atrioventricular (AV) block ranging from first- to third-degree is the most frequent presentation and may develop abruptly, posing a potentially fatal risk if not treated promptly [5,7,8]. Ventricular tachyarrhythmias, sinus node dysfunction, and the requirement for temporary pacing have also been reported [5,7].

There have been studies that describe cases of myocarditis, pericarditis, and myopericarditis that can mimic other infectious or inflammatory conditions and may present with transient ventricular dysfunction [2,3]. Endocardial involvement and aortitis have also been documented, including instances of endocarditis with positive molecular tests for *Borrelia* despite negative blood cultures [4,9,10]. Together, these findings demonstrate that *B. burgdorferi* can affect all cardiac layers: endocardium, myocardium, and pericardium. Therefore, a comprehensive synthesis of the available evidence is needed to better define clinical manifestations, improve diagnostic accuracy, and inform management strategies in this condition.

Despite these reports, the true incidence, full clinical spectrum, and optimal management strategies of cardiovascular involvement in Lyme disease remain incompletely characterized, with evidence largely derived from heterogeneous case reports and small series. Variability in diagnostic criteria and therapeutic approaches further contributes to clinical uncertainty.

Although uncommon, the cardiac complications of Lyme disease are clinically significant because they are often reversible with timely treatment. Management generally includes targeted antimicrobial therapy and, in selected cases, temporary pacemaker support or anti-inflammatory medication [2,3,5]. Most patients achieve a favorable outcome when treatment is initiated early; however, delayed diagnosis may adversely affect prognosis [2,6].

Given the heterogeneity in clinical presentation and the need for a specific diagnostic and therapeutic approach, this literature review aims to describe the cardiovascular manifestations documented in patients with Lyme disease, their clinical presentation, the treatment used, and the reported outcomes.

## 2. Materials and Methods

A narrative literature review was conducted to identify and summarize published evidence regarding cardiovascular manifestations of Lyme disease. Although this study was not designed as a formal systematic review, elements of structured search and selection methodologies, including principles from PRISMA, were considered to enhance transparency in study identification and selection.

Research sources: PubMed, MEDLINE, and Google Scholar.

Publication period: January 2000 to July 2025.

Languages: English and Spanish.

Search terms: Combinations of keywords and Boolean operators were used:

(“Lyme disease” OR “*Borrelia burgdorferi*”) AND (“carditis” OR “atrioventricularblock” OR “arrhythmia” OR “myocarditis” OR “pericarditis” OR “endocarditis” OR “aortitis”).

Inclusion criteria:

1—Case reports or case series with confirmed diagnosis of Lyme disease by serology, culture, or molecular testing.

2—Documentation of cardiovascular manifestations through clinical evaluation or invasive/non-invasive studies.

3—Available information regarding treatment and clinical outcomes.

Exclusion criteria:

1—Articles published prior to 2000.

2—Studies without confirmed Lyme disease diagnosis.

3—Reports lacking sufficient data on cardiovascular involvement.

Study selection process

All records were initially screened by title and abstract. Duplicate records identified across different databases (PubMed, MEDLINE, and Google Scholar), corresponding to the same publication retrieved from multiple sources, were removed manually.

Full-text articles were then assessed for eligibility according to predefined inclusion and exclusion criteria.

Original research articles, reviews, and guidelines without extractable individual patient-level data were excluded from the primary analysis. Only case reports and case series describing cardiovascular manifestations of Lyme disease with sufficient clinical detail (including presentation, diagnosis, management, and outcomes) were included in the final analysis.

**Figure 1:**
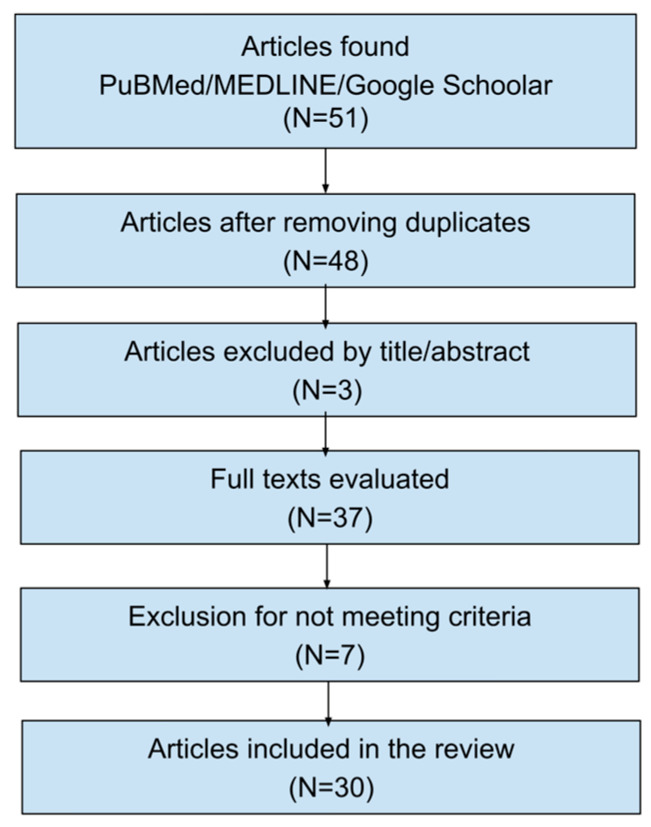
Flow diagram of the study selection process, including identification, screening, eligibility, and inclusion of articles.

Some reviews and broader original studies were retained as complementary references to support background information and discussion but were not included in the quantitative synthesis. The study selection process is summarized in Figure 1.

Bias and level of evidence:

No formal assessment of risk of bias or level of evidence (e.g., GRADE) was performed due to the narrative nature of the review. Most included studies correspond to case reports and small case series, representing a low level of evidence.

A total of 51 records were initially identified. After removal of duplicates and application of the inclusion and exclusion criteria, 30 studies were included in the final review.

## 3. Results

### 3.1. Atrioventricular (AV) Blocks

Atrioventricular (AV) blocks, particularly third-degree, represent the most frequently reported cardiovascular manifestation of Lyme disease [5,7,8]. Reported cases describe a wide spectrum of clinical presentations, ranging from asymptomatic conduction abnormalities to syncope and hemodynamic instability requiring urgent intervention.

Representative cases from the literature illustrate this variability. Patients may present with sudden syncope and complete AV block requiring temporary pacemaker placement, often in the context of recent tick exposure and compatible dermatologic findings. In other instances, conduction disturbances are identified through ambulatory monitoring, accompanied by arrhythmias such as supraventricular extrasystoles or bradycardia–tachycardia episodes, even in the absence of classic erythema migrans. Additionally, some patients exhibit multisystem involvement, with concurrent neurologic and cardiac manifestations, further complicating the clinical picture.

Overall, atrioventricular conduction disturbances represent the most consistent cardiac manifestation across the reviewed studies. Despite variability in presentation, most cases share common features, including acute onset, association with early disseminated infection, and favorable response to antimicrobial therapy. High-grade AV block frequently required temporary pacing; however, permanent pacemaker implantation was rarely necessary due to the reversible nature of the condition.

However, the available evidence is heterogeneous, with variability in diagnostic approaches, reporting detail, and clinical management across studies. Most data derive from case reports and small case series, which limits direct comparison and the ability to establish standardized patterns or draw definitive conclusions regarding prognosis and optimal management.

### 3.2. Carditis

Lyme carditis is an uncommon but potentially serious complication of Lyme disease, first described in 1980 and estimated to affect approximately 1.5–10% of infected patients, particularly those who are untreated or in later stages of disseminated disease [6,8]. Although relatively infrequent, its clinical relevance lies in its ability to impair cardiac conduction and, in severe cases, become life-threatening [3,6].

Epidemiologically, Lyme carditis occurs in 4–10% of patients with Lyme disease and is more frequently observed among young men aged 20–40 years, women aged 25–29 years, and adults older than 75 years [1,10]. Male sex has been identified as an independent risk factor, even in cohorts with a balanced sex distribution, with a reported male-to-female ratio of approximately 3:1 [3,9]. Interestingly, these sex-related differences do not appear to be associated with clothing habits, deodorant use, or outdoor activities at the time of tick exposure [9].

The pathophysiology of Lyme carditis involves infection of the cardiac tissue by *Borrelia burgdorferi*, triggering an exaggerated immune response that contributes to myocardial injury [10]. The bacterium employs surface proteins such as P66, which facilitate bacterial dissemination and confer a specific tropism for cardiac tissue [10]. In this setting, inflammation appears to play a key role in disease development, as antimicrobial therapy effectively treats the infection but may have a more limited impact on reversing cardiac manifestations [9].

Clinically, Lyme carditis presents with heterogeneous and variable symptoms: up to 30% of patients may remain asymptomatic, whereas others experience dizziness, syncope, dyspnea, palpitations, or chest pain [1,3]. Reported complications include sick sinus syndrome, atrial fibrillation, supraventricular and ventricular tachyarrhythmias, ventricular fibrillation, myocarditis, pericarditis, endocarditis, pericardial effusion, small-vessel vasculitis, and sudden cardiac death [1]. The most recognizable manifestation is atrioventricular block, which may require intravenous antimicrobial therapy and, in severe cases, temporary pacemaker support [6].

Diagnosis is challenging due to nonspecific clinical features. To aid evaluation, the Suspicious Index in Lyme Carditis (SILC) was developed. This scoring system incorporates demographic and clinical parameters such as male sex, age < 50 years, exposure to endemic areas, constitutional symptoms, tick bite, and erythema migrans, assigning up to 12 points and stratifying patients into low (0–2), intermediate (3–6), or high (7–11) risk for Lyme carditis [1,6,7]. In cases of unexplained arrhythmias or conduction abnormalities, the SILC score can be particularly helpful, especially when serologic testing is limited in acute presentations [7].

Diagnostic evaluation includes telemetry and cardiac imaging. Electrocardiography typically reveals conduction system impairment, whereas echocardiography may demonstrate pericardial effusion, systolic dysfunction, or cardiomegaly. For definitive diagnosis, cardiac magnetic resonance imaging or, in select cases, endomyocardial biopsy may be required [3].

Treatment guidance remains limited and heterogeneous; however, current recommendations include oral doxycycline or amoxicillin for stable patients, and intravenous ceftriaxone for those with hemodynamic compromise or systemic involvement, typically for 14–21 days [3]. Other effective antimicrobial options include cefuroxime and azithromycin [10]. The clinical frequency of Lyme carditis appears to be decreasing, likely due to earlier recognition and widespread antimicrobial use in early disease [6].

Although Lyme carditis is a well-recognized manifestation, some variability in presentation and diagnosis persists. Differences in diagnostic criteria, reliance on serological testing, and the absence of standardized definitions in some reports may affect the consistency of case classification. Furthermore, while conduction disturbances are strongly associated with *Borrelia burgdorferi*, caution is warranted when interpreting less typical manifestations, particularly when based on limited or isolated reports.

### 3.3. Endocarditis

Within this clinical spectrum, isolated valvular endocarditis associated with *Borrelia burgdorferi* infection is extremely rare, with polymerase chain reaction (PCR) confirmation reported only in a small number of cases [2].

Given its rarity and lack of specific clinical features, *B. burgdorferi* should be considered in the differential diagnosis of culture negative endocarditis, particularly when valvular pathology exhibits atypical features not attributed to conventional etiologic agents. In such cases, submission of valvular tissue for PCR-based molecular analysis is essential to confirm infection and establish a definitive diagnosis [2].

It is important to interpret these findings with caution. Most of the available evidence is derived from isolated case reports and small case series, which represent a low level of evidence and limit the ability to establish a causal relationship between *Borrelia burgdorferi* infection and endocardial involvement. In several cases, the diagnosis relies primarily on serological findings without definitive microbiological confirmation in valvular tissue, raising the possibility of coincidental association rather than true causality. Additionally, variability in diagnostic criteria and the presence of potential confounding factors further complicate the interpretation of these findings.

### 3.4. Myocarditis

Myocarditis associated with Lyme disease is an uncommon manifestation within the spectrum of cardiac involvement [4]. Myocardial infection by *Borrelia burgdorferi* typically occurs one to two months after disease onset. Although endomyocardial biopsy can provide diagnostic confirmation, its invasive nature and the risk of false-negative results due to the patchy distribution of inflammation limit its clinical utility and render it primarily of academic value [1,4].

The pathophysiology of Lyme myocarditis involves both direct invasion of myocardial tissue, including the atrioventricular node, and an exaggerated immune response. This inflammatory reaction, predominantly macrophage- and lymphocyte-mediated, is accompanied by cytokine release and evidence of cross-reactivity between anti-*Borrelia* antibodies and cardiac structures, supporting an autoimmune component in myocardial injury [3,8,10].

Clinically, Lyme carditis may manifest with infranodal block, ventricular or supraventricular arrhythmias, and ST segment or T wave abnormalities on electrocardiography, all of which reflect active myocardial involvement. Although most cases of Lyme myopericarditis are asymptomatic, some patients may present with symptoms mimicking acute coronary syndrome, complicating the diagnostic process [3,7]. Early clinical follow up is crucial, as sudden cardiac arrest in young patients may result from myocarditis, cardiomyopathies, or channelopathies. In this context, progressive normalization of electrocardiographic abnormalities can guide management, and in selected cases may justify obtaining a three generation pedigree or performing genetic testing [7].

Among noninvasive diagnostic tools, cardiac magnetic resonance imaging has demonstrated value both in confirmation and prognostic stratification. This modality can identify myocardial inflammation triggered by spirochetal infection, as well as pericardial enhancement suggestive of associated pericarditis [3].

### 3.5. Pericarditis

Pericarditis is an uncommon manifestation of *Borrelia burgdorferi* infection. Most reported cases correspond to asymptomatic myopericarditis; however, some patients may develop symptoms that mimic acute coronary syndrome, posing a diagnostic challenge in clinical practice [3].

Despite these atypical presentations, the overall prognosis of Lyme pericarditis is favorable. When clinical suspicion exists, antimicrobial treatment should be initiated promptly even before serologic confirmation to prevent complications and ensure early recovery. Based on case reports and expert opinion, the recommended regimen is intravenous ceftriaxone at 2 g once daily in adults, or 50–75 mg/kg/day in pediatric patients [3].

### 3.6. Aortitis

Aortitis is an uncommon inflammatory condition of the aortic wall, and infectious forms are typically associated with atherosclerotic aortic disease or with endocarditis [11]. Although aortitis-induced aneurysm formation is rare, an infected aorta may evolve into an aneurysm, and initial symptoms are often nonspecific such as fever, anorexia, weight loss, or abdominal pain until aneurysmal expansion or rupture occurs, leading to hemorrhagic and/or septic shock [11,12]. Histologically, aortitis is characterized by medial dissection and a predominantly neutrophilic inflammatory infiltrate without giant cells. Diagnostic workup typically begins with transesophageal echocardiography to rule out endocarditis and evaluate the thoracic aorta, with invasive aortography reserved for inconclusive cases.

Infectious aortitis carries a high mortality rate, particularly when associated with aneurysmal rupture. Poor prognostic factors include advanced age, delayed diagnosis, immunosuppression, thoracic involvement, medical-only treatment, and severe complications [12]. When infectious aortitis is suspected, broad-spectrum antimicrobial therapy and early vascular surgery consultation are essential [11].

### 3.7. Valvular Involvement

Valvular involvement in Lyme disease is exceedingly rare within the cardiovascular spectrum of *Borrelia burgdorferi* infection. Although conduction disturbances and carditis remain the predominant forms of cardiac involvement, isolated cases of valvular endocarditis confirmed by bacterial DNA in excised tissue have been reported [13,14,15,16].

Hidri et al. (2012) described one of the most notable cases, in which *B. burgdorferi* infection was confirmed by PCR in surgically excised mitral valve tissue, underscoring the diagnostic value of molecular testing in culture negative endocarditis [15]. Similarly, Fatima et al. (2018) and Haddad et al. (2019) reported mitral endocarditis with severe valvular insufficiency and vegetations, where histopathology revealed active inflammation without Aschoff bodies or granulomas, ruling out rheumatic or tuberculous etiologies [13,16].

Overall, these reports demonstrate that Lyme endocarditis may occur even in patients without apparent tick exposure or residence in endemic areas, highlighting the need for a high index of suspicion when evaluating culture-negative endocarditis or atypical valvular degeneration [14,16]. Culture of *Borrelia* from valvular tissue is typically unsuccessful due to the organism’s fastidious nature; however, universal bacterial PCR and specific serologic testing serve as valuable complementary diagnostic tools [17]. Collectively, the literature indicates that although rare, *B. burgdorferi* infection can alter valvular architecture, leading to severe regurgitation requiring surgical intervention, with generally favorable outcomes following targeted antimicrobial therapy and valve repair or replacement [13,14,15,16].

### 3.8. Vasculitis

Vascular manifestations in Lyme disease are rare. Vasculitis associated with *Borrelia burgdorferi* has been reported in approximately 0.3–1% of cases, while cerebrovascular accidents (CVAs) secondary to infection occur in <1% [15]. Vasculitis is generally considered the underlying mechanism responsible for ischemic stroke in the setting of neuroborreliosis [18].

Reported complications include cerebral ischemia, dural venous sinus thrombosis, subarachnoid hemorrhage, and intracerebral hemorrhage. Lyme vasculitis may affect vessels of large and medium caliber [17], causing stenosis, dilation, and contrast enhancement on imaging studies. Pathophysiologically, *B. burgdorferi* can cross the blood–brain barrier, invade the vascular wall, and induce inflammation through endothelial activation and release of pro-inflammatory mediators [18,19].

One case described a 58-year-old man [18] with no cardiovascular risk factors who presented with multiple cerebrovascular events. Magnetic resonance imaging revealed two cortical infarcts in the middle cerebral artery territory (parieto-occipital and frontal lobes). CT angiography showed no significant stenosis or occlusion. Extensive immunologic and serologic testing (including JAK2, ANCA, cryoglobulins, hepatitis, HIV, syphilis, tuberculosis, among others) yielded no significant findings. However, a history of a tick bite two years earlier prompted testing for Lyme disease, with serology and Western blot confirming infection. Notably, the patient had never exhibited erythema migrans or other classic Lyme manifestations. After initiation of ceftriaxone at recommended doses, he experienced complete clinical resolution without neurological sequelae [18].

This case highlights that rare manifestations such as Lyme vasculitis may occur even in the absence of typical clinical clues (e.g., clear tick bite, erythema migrans, or cranial neuritis), and that cerebrospinal fluid may be normal, complicating diagnosis [17,18,19].

Current evidence is largely limited to isolated case reports, representing a low level of evidence. In many cases, the diagnosis is based on indirect findings, such as imaging and serology, without histopathological confirmation, which limits the ability to establish a definitive causal relationship. The presence of other inflammatory or infectious conditions may also act as confounding factors. Therefore, these manifestations should be considered rare and not yet fully characterized complications of Lyme disease.

In order to facilitate a structured interpretation of the heterogeneous evidence, reported cases were systematically compiled and categorized based on clinical manifestations, diagnostic methods, management strategies, and outcomes. This approach enables the identification of consistent patterns as well as less frequent or atypical presentations within the cardiovascular spectrum of Lyme disease. The synthesized data are summarized in Table 1, with comparative analyses presented in Table 2.

## 4. Discussion

Cardiovascular involvement secondary to *Borrelia burgdorferi* infection is an uncommon yet clinically relevant manifestation within the spectrum of Lyme disease. Although its overall incidence is estimated at 1.5% to 10% of patients, timely recognition is essential due to the high rate of reversibility with early antimicrobial therapy [1,5,6].

Atrioventricular (AV) conduction disturbances remain consistently predominant over the years, with a frequency of 61.8% (Table 3). This finding reinforces that the cardiac conduction system is the primary target of *B. burgdorferi*, consistent with previous reviews.

Although less common, arrhythmias (32.4%) represented an important cause of clinical presentation, often prompting emergency evaluation and the performance of electrocardiographic studies, cardiac biomarkers, and even imaging modalities.

Similarly, myocarditis and myopericarditis were observed in nearly one-third of the reports, with favorable outcomes after antimicrobial therapy and hemodynamic support. These observations underscore the importance of considering Lyme disease in the differential diagnosis of acute myocarditis, particularly in endemic areas, an essential aspect in case reports without a typical early presentation.

In recent years, an increased number of vascular and large vessel manifestations such as vasculitis and aortitis has been documented, 0% in 2000–2019 (Table 4) vs. 16.7% in 2020–2025 (Table 5). This trend may reflect heightened interest in reporting atypical presentations, as well as the broader availability of advanced imaging techniques, including CT and MR angiography, which have facilitated the detection of *B. burgdorferi* involvement in vascular or valvular structures.

Valvular involvement and endocarditis were reported in 23.5% of cases, typically diagnosed as such through histopathological or molecular analysis following valve surgery. In these scenarios, establishing an etiologic diagnosis should not delay initial treatment, which may include valvular repair or replacement, followed by targeted antimicrobial therapy once the causative agent is identified.

An important consideration is the potential impact of bias on the findings of this review. A substantial proportion of the included evidence derives from case reports and small case series, which are inherently subject to publication and reporting bias. There is a tendency to preferentially report severe, atypical, or clinically striking presentations, potentially leading to an overrepresentation of uncommon manifestations such as valvular involvement, vasculitis, or aortitis. Conversely, milder or self-limited cases may be underreported. Additionally, variability in diagnostic approaches and reporting standards across studies may contribute to inconsistencies in the classification and interpretation of cardiovascular manifestations.

Geographical variability also represents an important limitation for generalizability. Most available studies originate from endemic regions in North America and Europe, where differences in *Borrelia* genospecies distribution, healthcare access, and diagnostic awareness may influence both the clinical spectrum and the likelihood of diagnosis. Therefore, these findings should be interpreted with caution when extrapolated to non-endemic or underrepresented regions.

Antimicrobial management across the reviewed cases aligns with the 2020 Clinical Practice Guidelines jointly issued by the Infectious Diseases Society of America (IDSA), the American Academy of Neurology (AAN), and the American College of Rheumatology (ACR) [34]. Most patients received intravenous ceftriaxone with transition to oral doxycycline at discharge; isolated use of amoxicillin plus gentamicin was reported in one case. The guidelines recommend ceftriaxone as the first-line agent, reserving oral options such as doxycycline, amoxicillin, cefuroxime, or azithromycin for mild presentations.

Regarding supportive care, temporary pacemaker placement was required in most cases. The guidelines discourage permanent pacemaker implantation, given that AV block typically resolves after bacterial eradication.

Most reports describe complete clinical resolution following appropriate treatment and supportive measures, including in complex cases. This reinforces that despite the potential severity of these presentations, early diagnosis and correct management have a decisive impact on prognosis.

It should be noted that this review also included systematic reviews and meta-analyses, which report multiple categories of manifestations and therefore increase the number of mentions within several groups. Classification depended on the descriptions provided by the authors; in some instances, distinctions between myocarditis and myopericarditis, or between valvular lesions and endocarditis, were not fully consistent.

## 5. Conclusions

Collectively, the findings from this review confirm that cardiac involvement due to *Borrelia burgdorferi* remains predominantly centered on conduction disturbances. However, the clinical spectrum has broadened to include less common forms involving the myocardium, pericardium, cardiac valves, and large vessels. Recognizing these variants and applying appropriate diagnostic strategies are essential to optimizing treatment and improving outcomes.

This review has several limitations. The included studies are heterogeneous and largely based on case reports and small series, which limits generalizability. The inclusion of systematic reviews alongside primary studies may have led to overrepresentation of certain manifestations. In addition, variability in diagnostic criteria across studies may have affected classification consistency.

Future research should focus on large prospective studies and standardized diagnostic approaches to better define the incidence, clinical spectrum, and optimal management of cardiovascular involvement in Lyme disease.

## Figures and Tables

**Table 1:** Cases 2020–2025.

Article/Study Type	Manifestation	Key Findings	Diagnosis	Management	Outcome
Carnazzo 2023 et al. Case Report [1]	Complete AV block; MR/TR	Syncope; erythema migrans	ELISA; Western blot	TPM + ceftriaxone + doxycycline	Complete resolution
Najam 2023 et al. Case Report [20]	Bradycardia; 1st AV block; Mobitz I; 2:1 AV block	calcified aortic stenosis; orthopnea; dyspnea	Serology + clinical	Ceftriaxone + doxycycline	Complete resolution
Javed et al. 2024 et al. Systematic review [9]	Analyzed 110 cases; >78% male; 40% Manifestation: AV block predominance (110 cases) Key findings; syncope 29%; palpitations 13%; stable majority (104) 55% no echocardiographic findings		Serology + clinical	antimicrobials; 63.33% TPM 20%	80% resolved 6 deaths
Esfandiari 2022 et al. Case report [7]	Polymorphic VT; cardiac arrest; myocarditis	myocardial edema	Serology + Western blot	CPR/epinephrine + ceftriaxone + doxycycline	Complete resolution
Ameer 2024 et al. Case report [3]	Chest pain; tachycardia	ST elevation; PR depression; pericarditis	Clinical (Pericarditis induced by Lyme)	Aspirin + colchicine + doxycycline.	Unknown
Lytvyn 2021 et al. Case report [5]	Tachycardi; 1st AV block; ventricular ectopy	Myocarditis	Serology + Western blot	Doxycycline	Complete resolution
Schick et al. 2020 et al. Case report [4]	Sinus tachycardia; left jaw; LVEF 56%; bradycardia; AV block progression	**↑** troponins; **↑** BNP; lymphadenopathy	serology	Doxycycline + ceftriaxone	Complete resolution
Zaid 2022 et al. Case report [8]	Bradycardia; 1st AV block; RBBB	Carditis	serlogy + lumbar puncture	Ceftriaxone	Complete resolution
Gomez-Tschrnk 2024 et al Case report [2]	Mitral insufficiency with vegetation; bicuspid calcified aortic valve with insufficiency and perforation; LVEF 55%	Headache; arm paresis; subarachnoid hemorrhage	valve tissue sequencing + serology	Doxycycline	Complete resolution
Khetpal et al. 2021 et al. Case report [6]	Complete AV block; Lyme carditis	Fatigue; dizziness, dyspnea; erythema migrans	serology + Western blot	Doxycycline	Complete resolution
Batikyan 2025. Case report [10]	Fatigue; weaken; palpitations, presyncope; dyspnea	Bradycardia; pulmonary congestion	serology + Western blot	TPM + ceftriaxone + doxycycline	Complete resolution
Correia et al. 2020 et al. Case report [12]	General malaise; **↑** leukocytosis; **↑** ESR; **↑** PCR	Oppressive precordial pain; aortitis (diffuse thickening of arch and thoracic aorta)	serology	Doxycycline	Aortic dissection (Lyme vasculitis) with surgical correction
Isha, S et al. 2023 Case report [21]	Bradycardia; complete AV block	Syncope; **↑** NT-proBNP;	Serology	TPM + Ceftriaxone	Complete resolution
Uzomah et al. 2021 Retrospective cohort [22]	It seeks to identify incidence and predictive factors for permanent pacemaker implantation in patients Manifestation: AV blocks; sinoatrial dysfunction Kye findings: 11% Lyme disease developed carditis		not specified	2% TPM (287/9729)	hospital duration was 5.2 days and 1.5%. mortality
Myers 2020 et al. Case report [23]	Chest pain; 1st AV block; sinus pauses	Erythema migrans	Serology	Ceftriaxone + doxycycline	Complete resolution
Grewal 2023. Case report [24]	Complete heart block; RBBB	AV block	Serology	Ceftriaxone + permanent pacemaker	Complete resolution
Tabot 2023. Case report [25]	Palpitations; dyspnea; chest pain	LBBB	Serology	Ceftriaxone	Complete resolution
Malik et al. 2021 Case report [26]	Oppressive chest pain; ST elevation; LVEF 49%	Diaphoresis; tachycardia	Serology	Heparin (suspected ACS) + ceftriaxone + doxycycline	Complete resolution
Riescher 2023 et al. Case report [18]	Asthenia; dizziness; paresthesia in right arm	Neurologic symptoms (cryptogenic stroke); Interatrial septal aneurysm	Serology + Western blot	Aspirin + atorvastatin + perindopril	Complete resolution
Kaldas 2025 et al. Case report [27]	Erythema migrans; ACS; Lyme carditis; cardiogenic shock; LVEF 15%	Cardiac arrest due to AF; **↑** troponin; anterolateral ischemia; + 80% stenosis of the internal descending artery	Serology	cardioversions + dobutamine + amiodarone + doxycycline + ceftriaxone + stent placement	ICD placed for secondary prevention

AV—Atrioventricular; ELISA—Enzyme-Linked Immunosorbent Assay; VT—ventricular tachycardia; LVEF—left ventricular ejection fraction; BNP—B-type natriuretic peptide; NT-proBNP—N-terminal pro-B-type natriuretic peptide; RBBB—right bundle branch block; PCR—polymerase chain reaction. ESR—erythrocyte sedimentation rate; LBBB—Left bundle branch block; ACS—Acute coronary syndrome; AF—atrial fibrillation. ICD—implantable cardioverter defibrillator; MR—mitral regurgitation; TR—tricuspid; **↑**—elevation TPM—Temporary pacemaker.

**Table 2:** Cases 2000–2020.

Article/Study Type	Manifestation	Key Findings	Diagnosis	Management	Outcome
Chaus 2018 et al. Case report [28]	Left-eye amyloidosis history	Syncope; Bradycardia; complete AV block; paroxysmal flutter	serology	Ceftriaxone	1st AV block
Koene 2012 et al. Case report [29]	Erythema migrans	Complete AV Block; polymorphic VT; severe biventricular failure (LVEF <10%)	Serology + Western blot	Methylprednisolo ne + TPM + ceftriaxone + doxycycline	Complete resolution
Steere et al. Case series [30]	20 patients(ages 6–58) 95% erythema migrans 90%AV block (40% complete block) 65% ECG changes compatible with myopericarditis 65% with polyarteritis 20% with LVEF 40%		Serology	9/20 prednisone 6/20 TPM 8/20 penicillin	No deaths; complete recovery
Brownstein 2016 et al Case report [31]	Anxiety; Depression; insomnia	Syncope; 2nd AV block; 5–6 s asystole	Serology	TPM + doxycycline	Complete Resolution (Pacemaker removed at 3 months)
Zainal 2019 Case report [32]	Diaphoresis; arthritis; erythema migrans	Chest pain; Dyspnea; AF; 1st AV block	C6 peptide and Lyme positive + Western blot	Ceftriaxone	Complete resolution
Fatima 2018 et al. Case report [13]	Dyspnea NYHA IV; bilateral pleural effusion.	Recurrent Lyme Carditis; AF; **↑** BNP; severe mitral insufficiency with perforation; tricuspid insufficiency	Borrelia DNA from mitral valve tissue + Serology	Mitral valve repair + aortic valve replacement + tricuspid repair + ceftriaxone q	Complete resolution
Gilson 2017 et al. Case report [33]	Headache seconary to sinusitis; facial paralysis	Chest pain; troponin 7.82 + CK-MB 75.7	Serology	Catheterization + ceftriaxone + steroids	Complete resolution
Canver 2000 et al. Case report [14]	Chest pain; dyspnea; palpitations	1st AV Block; severe mitral insufficiency; pericardial effusion	Serology + Western blot	Mitral valve replacement + anterior thoracotomy for tamponade	Complete resolution
Hidri 2012 et al. Case report [15]	History of paroymal AF; mitral insufficiency due to valve prolapse and perforation	Echo: LVEF 45%; LA dilation; intraoperative diagnosis of endocarditis	PCR on mitral valve + serology + Western blot	Mitral valve replacement + amoxicillin + gentamicin	Complete resolution
Haddad 2019 et al. Case report [16]	Progressive fatigue; dyspnea; degenerative mitral disease	Echo with severe mitral insufficiency due to anterior leaflet prolapse with perforation	Mitral valve PCR + serology	Valve repair + ceftriaxone	Complete resolution

AV—Atrioventricular; VT—ventricular tachycardia; LVEF—left ventricular ejection fraction; CK-MB—Creatine kinase-MB; PCR—polymerase chain reaction. ECG—Electrocardiogram AF—atrial fibrillation. NYHA—New York Heart Association DNA—deoxyribonucleic acid. **↑**—elevation.

**Table 3:** Frequency of reported cardiovascular manifestations in Lyme disease based on the reviewed literature (2000–2025).

Cardiovascular Manifestation	N of Articles (Multiple Count)	Overall (%)
AV block/conduction disorders	21	61.8%
Arrhythmias (AF, VT, others)	11	32.4%
Myocarditis	10	29.4%
Endocarditis/valvulopathy	8	23.5%
Vasculitis	7	20.6%
Pericarditis	6	17.6%
Aortitis	4	11.8%

Note: A total of 30 studies were included. Each study was counted in every cardiovascular category it reported (multiple counting). The reported proportions reflect frequency of mentions within the literature and should not be interpreted as population-based prevalence. Most data derive from case reports and small case series.

**Table 4:** Table for the period 2000–2019; 10 articles.

Cardiovascular Manifestation	N of Articles (Multiple Count)	2000–2020
AV block/conduction disorders	6	60.0%
Arrhythmias (AF, VT, others)	3	30.0%
Myocarditis	3	30.0%
Endocarditis/valvulopathy	4	40.0%
Vasculitis	2	20.0%
Pericarditis	1	10.0%
Aortitis	0	0%

**Table 5:** Table for the period 2020–2025; 20 articles.

Cardiovascular Manifestation	N of Articles (Multiple Count)	2000–2020
AV block/conduction disorders	15	62.5%
Arrhythmias (AF, VT, others)	8	33.3%
Myocarditis	7	29.2%
Endocarditis/valvulopathy	4	16.7%
Vasculitis	4	25.0%
Pericarditis	6	16.7%
Aortitis	4	16.7%

## Data Availability

No new data were created or analyzed in this study.
